# Investigation of optimal gestational weight gain based on the occurrence of adverse pregnancy outcomes for Chinese women: a prospective cohort study

**DOI:** 10.1186/s12958-021-00797-y

**Published:** 2021-08-30

**Authors:** Yin Sun, Zhongzhou Shen, Yongle Zhan, Yawen Wang, Shuai Ma, Suhan Zhang, Juntao Liu, Sansan Wu, Yahui Feng, Yunli Chen, Shuya Cai, Yingjie Shi, Liangkun Ma, Yu Jiang

**Affiliations:** 1 Department of Obstetrics and Gynecology, Peking Union Medical College Hospital, Chinese Academy of Medical Sciences & Peking Union Medical College, National Clinical Research Center for Obstetric & Gynecologic Diseases, No. 1 Shuaifuyuan Wangfujing, Dongcheng District, Beijng, 100730 China; 2 School of Public Health, Chinese Academy of Medical Sciences and Peking Union Medical College, No. 9 Dongdan Santiao, Dongcheng District, Beijing, 100730 China

**Keywords:** Chinese pregnant women, Gestational weight gain, Recommendations, Pre-pregnancy BMI, Cohort study

## Abstract

**Objective:**

To investigate recommendations for appropriate gestational weight gain (GWG) of Chinese females.

**Methods:**

In total of 3,172 eligible women in the first trimester were recruited into the Chinese Pregnant Women Cohort Study (CPWCS) project. Pregnancy complications and outcomes were collated using the hospital medical records system. The method of occurrence of participants with adverse pregnancy outcomes (Occurrence Method) was conducted to calculate the recommended total GWG for each participant’s pre-pregnancy BMI. Occurrence Method data were judged against the Institute of Medicine (IOM) and Japanese recommended criteria in terms of the total occurrence of adverse pregnancy outcomes of pregnant women with appropriate weight gain.

**Results:**

The most frequent GWG was ≥ 14 kg and < 16 kg (19.4%), followed by ≥ 10 kg and < 12 kg (15.5%) and ≥ 12 kg and < 14 kg (15.2%). The most frequently occurring adverse pregnancy outcomes were cesarean sections for underweight (30.0%), normal weight (40.4%), overweight (53.6%) and obese (53.7%) women. A large for gestational age (LGA) accounted for 18.0% of the overweight and 20.9% of the obesity group. Gestational diabetes mellitus (GDM) occurred in 16.9% of overweight and 23.1% of obese women. The recommended total GWG in a Chinese women population is ≥ 8 and < 12 kg if underweight, ≥ 12 and < 14 kg for normal weight, ≥ 8.0 and < 10.0 kg if overweight, and < 8 kg for women with obesity.

**Conclusions:**

Current Chinese recommendations provide the optimal ranges of GWG to minimize the occurrence of undesirable pregnancy outcomes for each group of pre-pregnancy BMIs in a Chinese population.

**Trial registration:**

Registered with ClinicalTrials (NCT03403543).

**Supplementary Information:**

The online version contains supplementary material available at 10.1186/s12958-021-00797-y.

## Background

Prenatal and postnatal care are important healthcare issues in most countries and how to improve mothers, fetus and child healthcare has become an important health goal worldwide [1]. Evidence suggests that gestational weight gain (GWG) is directly associated with mother and child health outcomes, and has a significant impact on pregnancy complications and outcomes [2, 3]. Meta-analysis of 39 cohorts of mothers showed that about 32% of large for gestational age (LGA) babies could be accounted for by too much GWG [4]. In contrast, women who did not gain adequate weight had higher probabilities of experiencing anemia [5], preterm birth (PB) [3], and delivering low birth weight (LBW) [6] and small for gestational age (SGA) infants [7], while participants who gained excessive weight had a greater risk of having gestational diabetes mellitus (GDM) [8], gestational hypertension (GH) [9], pre-eclampsia [5] and the need for caesarean sections [6].

However, studies indicated that the occurrence of inappropriate GWG is common worldwide. Shulman et al. found that 27.0% of pregnant women did not acquire accurate knowledge of weight gain recommendations and 30.0% did not have appropriate GWG during pregnancy in Atlanta [10]. Vivatkusol et al. pointed out that the prevalence of inappropriate GWG approached 61.7% in Thailand [5] and Shao and colleagues reported that the incidence of appropriate GWG was only 25.9% in Chinese women [11].

Although the Institute of Medicine (IOM) has published recommended criteria for appropriate weight gain during pregnancy [12], a recent US study concluded that 73% of pregnancies had GWG above 2009 IOM guidelines [13]. The recommendations cannot be readily applied in different countries mainly due to different ethnicities and diets [14]. Jiang et al. [15] pointed out that the IOM recommended GWG ranges were likely too great for pregnant Chinese women classified according to their BMIs. Thus, specific recommendations should be defined for particular ethnic populations having significantly different maternal anthropometry.

Recent studies [16–19] have concentrated on investigations into recommended GWG for Chinese women. The Obstetrics and Gynecology Branch of the Chinese Medical Association put forward recommended weight gains of 12.5 – 18.0 kg for underweight women, 11.5 – 16.0 kg for normal weight, 7.0 – 11.5 kg for women who are moderately overweight and 5.0 – 9.0 kg for obese women [20]. Wang et al. considered an average GWG of 16 kg to be optimal following research into infant birth weight [21]. However, there are several limitations to these studies. First, most of them were retrospective and lacked a reliable inference of causality. Second, most studies selected women undergoing a normal pregnancy to calculate the recommendations, so the findings may not be applicable in clinical practice due to selection bias and lack of sufficient rigorous information.

The Chinese Pregnant Women Cohort Study-Peking Union Medical College (CPWCS-PUMC) is a multicenter, prospective and ongoing cohort study, which was established to provide relevant scientific evidence to guide the healthcare of pregnant Chinese women. In the present study, pregnant women from the CPWCS-PUMC in their first trimester were selected as subjects. We aim to investigate the proper recommended weight gain for Chinese women in different pre-pregnancy women allocated to according to their BMIs. The findings will be important for exploring the appropriate recommended GWG for a Chinese population, and should be of great help in developing suitable recommended criteria for clinical guidance in mainland China.

## Methods

### Setting and participants

This study was based on CPWCS-PUMC population research from 24 hospitals in 15 provinces of China from 2017 to 2018. Pregnant women in their first trimester were selected as subjects with the following inclusion criteria: (1) Chinese nationality (2) ≥ 16 years old; (3) 5 ~ 12 weeks’ gestational age; (4) permanent residents in the study locations; (5) willing to sign written informed consent. Exclusion criteria were: (1) pregnancy > 12 gestational weeks; (2) no regular birth inspection; (3) floating population; (4) those who have contraindications to pregnancy such as gynecological tumors. The study protocol was registered at Clinical Trials (NCT03403543) and approved by the Ethics Review Committee of Peking Union Medical College Hospital (HS-1345). Written informed consent was obtained from all participants prior to enrollment.

### Recruitment

The baseline sample of CPWCS included 7,976 women at the first trimester recruited between 25^th^ July 2017 and 24^th^ July 2018. Data on 3,767 pregnant women with singleton pregnancies were collected through 31^st^ December 2018. A total of 144 (3.8%) participants don’t have information on prenatal visits. Of the remaining 3,623 participants, 451 women without weight or height data measured during the first prenatal examination or weight just before delivery were excluded from the analysis, leaving a total of 3,172 eligible women. The detailed recruitment process can be seen in our previous paper [22].

### Measurements

#### BMI before pregnancy and GWG

BMI (kg/m^2^) values before pregnancy were calculated by measuring the height and weight of pregnant women at their first prenatal examination (5 ~ 12 weeks’ gestational age). Pre-pregnancy BMI was classified by closely following the "Adult Weight Determination" classification of the health industry standard (WS/T 428–2013) [23]. The categories were: underweight, BMI < 18.5 kg/m^2^; normal weight 18.5 ≤ BMI < 24.0 kg/m^2^; overweight 24.0 ≤ BMI < 28.0 kg/m^2^; and obese BMI ≥ 28.0 kg/m^2^. GWG was evaluated as the weight measured before delivery at the last prenatal examination subtracted from the weight measured at the initial prenatal examination and was obtained from the hospital electronic medical records system.

#### Number of women with pregnancy outcomes that were adverse

**Fig. 1:**
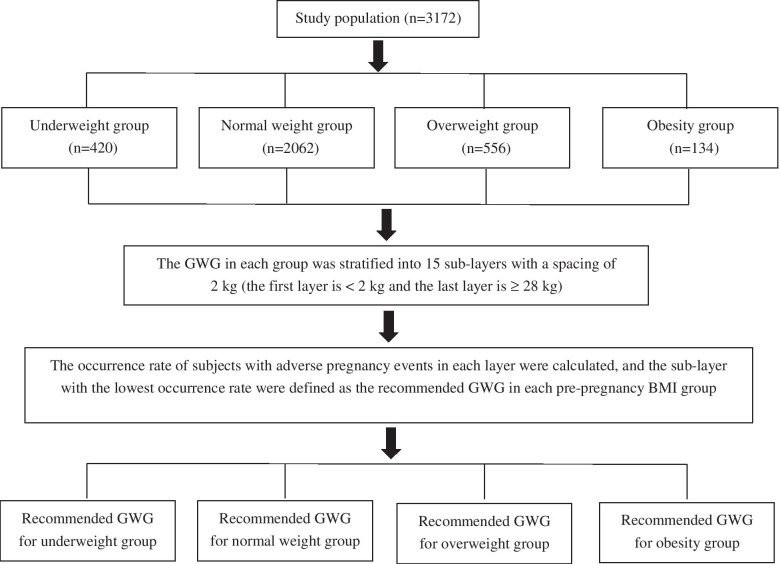
Flowchart of the Occurrence Method

The method of occurrence of participants with adverse pregnancy outcomes (Occurrence Method) was conducted to calculate the recommended GWG for each pre-pregnancy BMI group. Ten adverse pregnancy outcomes were included: gestational anemia (GA), GDM, GH (including preeclampsia), premature rupture of membranes (PROM), PB, LBW, macrosomia (MAC), SGA, LGA, and cesarean section (CS). As shown in Fig. 1, first, the women were assigned to 1 of 4 groups according to their BMI classification before becoming pregnant. Second, the GWG in each group was stratified into 15 sublayers with a spacing of 2 kg, and the occurrence of participants with adverse pregnancy outcomes in each layer was determined. Finally, the sublayer with the lowest occurrence of adverse outcomes was defined as the recommended GWG for each BMI group.

#### Evaluation of different recommendation criteria for GWG

**Fig. 2:**
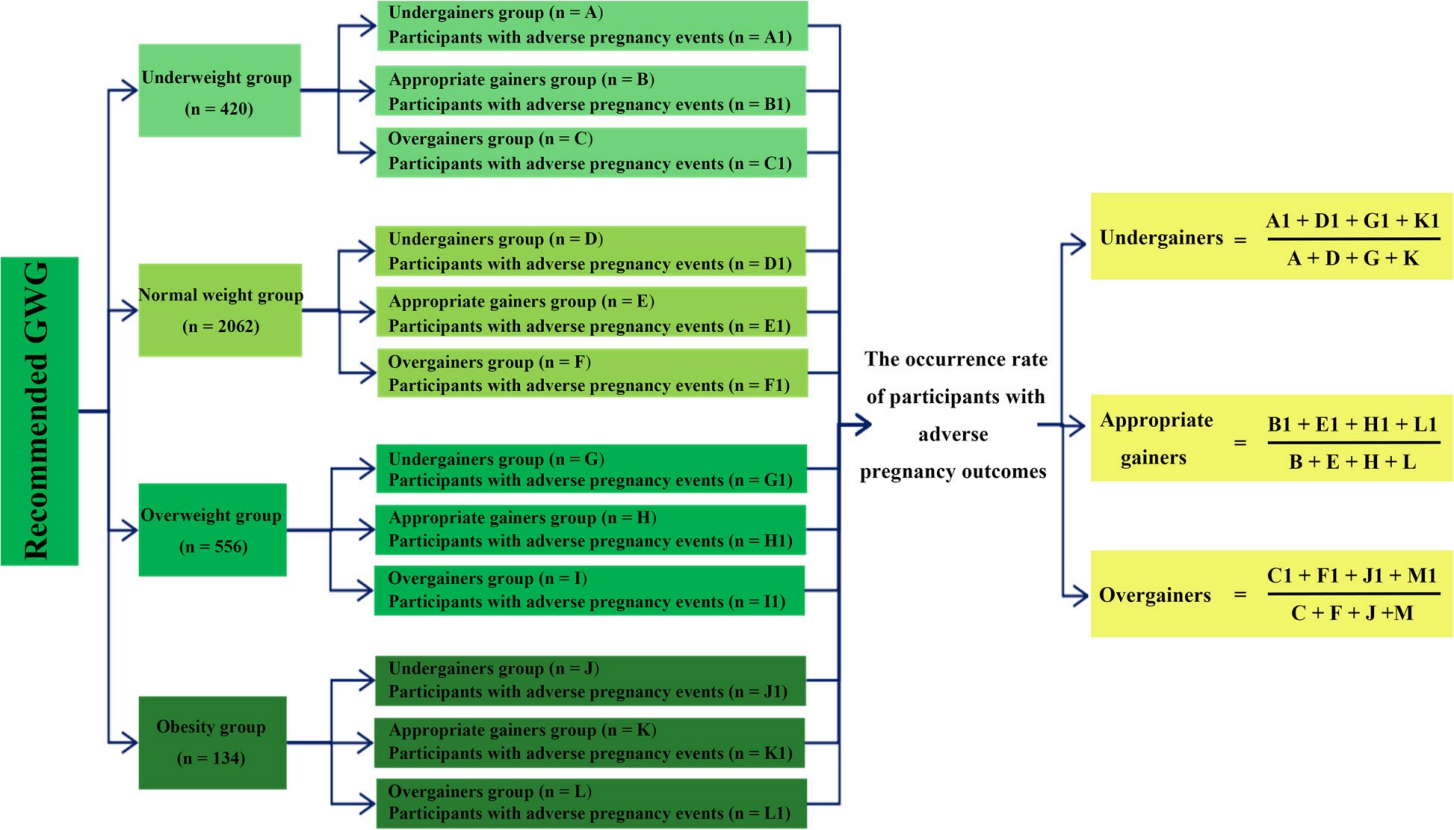
The algorithm of evaluation and comparison of different recommended GWGs. Note: GWG: gestational weight gain

In this study, we included three recommendation criteria for evaluation, namely IOM recommendations [24], Japan recommendations [25] and the China (Occurrence Method) recommendations of the present study. The United States IOM advises a GWG of 12.5 – 18 kg for women who are underweight, 11.5 – 16 kg for normal weight, 7 – 11.5 kg for overweight and 5 – 9 kg for obese women [26]. Japan defined GWG recommendations in 2001 [27] thus: 9 – 12 kg for women who are underweight, 7 – 12 kg for women of normal weight, ≤ 7 kg for overweight women and ≤ 5 kg for obese women. Women who put on less weight than recommended, the recommended weight gain, or more than the recommended weight gain were assigned to groups of undergainers, appropriate gainers and overgainers, respectively. In each category, pregnant women with different pre-pregnancy BMIs were pooled to calculate the overall occurrence of women with adverse pregnancy outcomes. By comparing the total incidence of pregnancy outcomes that were adverse in women who had appropriate weight gains, the lowest recommended value of the total occurrence of adverse pregnancy outcomes was determined as the appropriate recommended value of GWG in our study (Fig. 2).

### Definitions of adverse pregnancy outcomes

A diagnosis of GDM was made following the guidelines published by the American Diabetes Association in 2014 [28]. All the women were given an oral glucose (75 g) tolerance test (24 – 28 weeks gestation), and GDM was diagnosed if any of the following values was greater than or equal to: fasting glucose 5.1 mmol/L, glucose 10.0 mmol/L at 1 h, glucose 8.5 mmol/L at 2 h. According to the 8^th^ edition of Obstetrics and Gynecology [29] GH refers to systolic ≥ 140 mmHg or diastolic ≥ 90 mmHg (systolic ≥ 140 mmHg or diastolic ≥ 90 mmHg when reexamined at 4-h interval) after 20 weeks of gestation. 28 weeks ≤ gestational weeks < 37 weeks was considered as PB. Peripheral blood hemoglobin < 110 g/L and a hematocrit < 0.33 during pregnancy was defined as gestational anemia (GA). PROM was suspected based on symptoms and speculum examination and might have been supported by testing the vaginal fluid or by ultrasound [30]. Maternal adverse outcomes including CS, GA, PROM, GDM, GH and PB were collected by physicians at the 6-week postpartum follow-ups.

Neonates, with birth weights < 2,500 g were considered as LBWs and a weight ≥ 4,000 g as MAC. Neonates weights < the 10^th^ percentile and/or length ≥ 2 standard deviations below the mean for their gestational age were classified as SGA, while birth weights > the 90^th^ percentile and/or length ≥ 2 standard deviations > the mean for their gestational age as LGAs. Neonatal adverse outcomes including LBW, MAC, SGA and LGA were collected during physicians’ home visits to the mother’s home at the sixth week postpartum.

### Statistical analysis

Frequencies and percentages were calculated for categorical data. Data were collated and analyzed using Microsoft Office Excel 2013 and SPSS ver. 25.

## Results

### Pre-pregnancy BMIs and GWGs

**Fig. 3:**
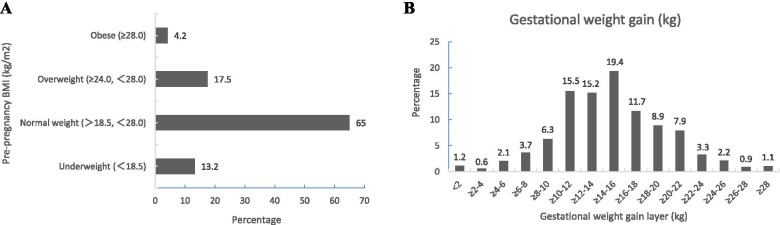
Distribution of pre-pregnancy BMI (**A**) and gestational weight gain (**B**) of the study population

Analysis of BMIs in pre-pregnant women and 15 GWG layer analysis of subjects revealed that pregnant women with a normal pre-pregnancy BMI accounted for the majority (65.01%), followed by overweight and obesity (21.75%) and low weight (13.24%) (Fig. 3A). The greatest gain in weight during pregnancy was ≥ 14.0 kg and < 16.0 kg, accounting for 19.4%, followed by ≥ 10.0 kg and < 12.0 kg (15.5%) and ≥ 12.0 kg and < 14.0 kg (15.2%) (Fig. 3B).

### Frequency distribution of adverse outcomes

The frequency of adverse pregnancy outcomes in different GWG sub-layers among the four pre-pregnant BMI groups is shown in Tables 1, 2, 3 and 4. The number of low-weight (82, 19.6%) and normal-weight (424, 20.6%) pregnant women who gained ≥ 14 kg and < 16 kg were the largest, and for overweight (94, 17.0%) and obese (30, 22.4%) women, a GWG ≥ 10 kg and < 12 kg was most common. In the four pre-pregnancy BMI groups, the most frequently occurring adverse pregnancy outcomes were CS (30.0, 40.4, 53.6 and 53.7%, respectively). In the overweight group, the three most common adverse outcomes, except for CS, were LGA (18.0%), GDM (16.9%) and PROM (16.0%). In the obesity group, the three most common adverse outcomes, except for CS, were GDM (23.1%), LGA (20.9%) and MAC (16.4%).

**Table 1:** Frequency (n, %) of adverse pregnancy outcomes in different GWG sublayers in the underweight group

	**Total subjects per sublayer**	**PB** **n (%)**	**LBW** **n (%)**	**MAC** **n (%)**	**SGA** **n (%)**	**LGA** **n (%)**	**CS** **n (%)**	**GA** **n (%)**	**PROM** **n (%)**	**GDM** **n (%)**	**GH** **n (%)**
** < 2**	1 (0.2)	1 (100)	1 (100)	0 (0)	0 (0)	0 (0)	0 (0)	0 (0)	0 (0)	0 (0)	0 (0)
** ≥ 2–4**	1 (0.2)	0 (0)	0 (0)	0 (0)	0 (0)	0 (0)	0 (0)	0 (0)	0 (0)	0 (0)	0 (0)
** ≥ 4–6**	5 (1.2)	1 (20.0)	1 (20.0)	0 (0)	0 (0)	0 (0)	2 (40.0)	1 (20.0)	1 (20.0)	1 (20)	0 (0)
** ≥ 6–8**	13 (3.1)	3 (23.1)	3 (23.1)	0 (0)	2 (15.4)	0 (0)	5 (38.5)	2 (15.4)	3 (23.1)	3 (23.1)	0 (0)
** ≥ 8–10**	21 (5.0)	3 (14.3)	2 (9.5)	0 (0)	2 (9.5)	0 (0)	6 (28.6)	0 (0)	1 (4.8)	1 (4.8)	0 (0)
** ≥ 10–12**	50 (11.9)	2 (4.0)	1 (2.0)	0 (0)	4 (8.0)	3 (6)	10 (20.0)	5 (10.0)	5 (10.0)	4 (8.0)	0 (0)
** ≥ 12–14**	58 (13.8)	3 (5.2)	3 (5.2)	1 (1.7)	5 (8.6)	4 (6.9)	13 (22.4)	4 (6.9)	7 (12.1)	6 (10.3)	0 (0)
** ≥ 14–16**	82 (19.6)	2 (2.4)	5 (6.0)	4 (4.8)	9 (10.8)	4 (4.8)	24 (28.9)	8 (9.6)	9 (10.8)	7 (8.4)	1 (1.2)
** ≥ 16–18**	64 (15.3)	6 (9.4)	3 (4.7)	0 (0)	2 (3.1)	1 (1.6)	23 (35.9)	13 (20.3)	8 (12.5)	4 (6.3)	3 (4.7)
** ≥ 18–20**	42 (10.0)	1 (2.4)	0 (0)	4 (9.5)	1 (2.4)	6 (14.3)	15 (35.7)	8 (19.0)	3 (7.1)	1 (2.4)	1 (2.4)
** ≥ 20–22**	36 (8.6)	0 (0)	0 (0)	3 (8.3)	3 (8.3)	3 (8.3)	13 (36.1)	4 (11.1)	2 (5.6)	2 (5.6)	0 (0)
** ≥ 22–24**	20 (4.8)	1 (5.0)	2 (10.0)	2 (10.0)	4 (20.0)	2 (10.0)	6 (30.0)	1 (5.0)	4 (20.0)	2 (10.0)	0 (0)
** ≥ 24–26**	14 (3.3)	0 (0)	0 (0)	0 (0)	0 (0)	0 (0)	5 (35.7)	2 (14.3)	3 (21.4)	0 (0)	0 (0)
** ≥ 26–28**	5 (1.2)	0 (0)	0 (0)	0 (0)	0 (0)	0 (0)	2 (40.0)	0 (0)	0 (0)	1 (20.0)	0 (0)
** ≥ 28**	7 (1.7)	0 (0)	0 (0)	2 (28.6)	0 (0)	2 (28.6)	2 (28.6)	2 (28.6)	1 (14.3)	1 (14.3)	0 (0)
**Total**	420	23 (5.5)	21 (5.0)	16 (3.8)	32 (7.6)	25 (6.0)	126 (30.0)	50 (11.9)	47 (11.2)	33 (7.9)	5 (1.2)

*CS* Cesarean section, *GA* Gestational anemia, *GDM* Gestational diabetes mellitus, *GH* Gestational hypertension, *LBW* Low birth weight, *LGA* Large for gestational age, *MAC* Macrosomia, *PB* Preterm birth, *PROM* Premature rupture of membranes, *SGA* Small for gestational age

**Table 2:** Frequency (n, %) of adverse pregnancy outcomes in different GWG sublayers in the normal weight group

	**Total subjects in sublayers**	**PB** **n (%)**	**LBW** **n (%)**	**MAC** **n (%)**	**SGA** **n (%)**	**LGA** **n (%)**	**CS** **n (%)**	**GA** **n (%)**	**PROM** **n (%)**	**GDM** **n (%)**	**GH** **n (%)**
** < 2**	14 (0.7)	0 (0)	2 (14.3)	0 (0)	5 (35.7)	0 (0)	7 (50.0)	2 (14.3)	2 (14.3)	1 (7.1)	0 (0)
** ≥ 2–4**	10 (0.5)	1 (10.0)	1 (10.0)	1 (10.0)	1 (10.0)	2 (20.0)	3 (30.0)	0 (0)	0 (0)	0 (0)	0 (0)
** ≥ 4–6**	38 (1.8)	5 (13.2)	1 (2.6)	0 (0)	4 (10.5)	3 (7.9)	12 (31.6)	4 (10.5)	5 (13.2)	10 (26.3)	0 (0)
** ≥ 6–8**	55 (2.7)	4 (7.3)	4 (7.3)	1 (1.8)	7 (12.7)	3 (5.5)	25 (45.5)	10 (18.2)	3 (5.5)	6 (10.9)	1 (1.8)
** ≥ 8–10**	123 (6.0)	7 (5.7)	6 (4.9)	6 (4.9)	4 (3.3)	8 (6.5)	51 (41.5)	18 (14.6)	19 (15.4)	25 (20.3)	2 (1.6)
** ≥ 10–12**	316 (15.3)	25 (7.9)	17 (5.4)	11 (3.5)	23 (7.3)	18 (5.7)	101 (31.9)	48 (15.1)	48 (15.1)	44 (13.9)	6 (1.9)
** ≥ 12–14**	328 (15.9)	12 (3.7)	12 (3.7)	15 (4.6)	25 (7.6)	26 (7.9)	120 (36.6)	46 (14.0)	41 (12.5)	30 (9.1)	6 (1.8)
** ≥ 14–16**	424 (20.6)	20 (4.7)	15 (3.5)	24 (5.7)	23 (5.4)	32 (7.5)	188 (44.3)	63 (14.9)	43 (10.1)	37 (8.7)	7 (1.7)
** ≥ 16–18**	240 (11.7)	4 (1.7)	5 (2.1)	14 (5.8)	16 (6.7)	22 (9.2)	90 (37.5)	44 (18.3)	26 (10.8)	19 (7.9)	6 (2.5)
** ≥ 18–20**	191 (9.3)	4 (2.1)	6 (3.1)	10 (5.2)	9 (4.7)	17 (8.9)	75 (39.3)	14 (7.3)	15 (7.9)	21 (11.0)	5 (2.6)
** ≥ 20–22**	163 (7.9)	3 (1.8)	3 (1.8)	13 (7.9)	7 (4.3)	23 (14.0)	77 (47.0)	25 (15.2)	31 (18.9)	11 (6.7)	4 (2.4)
** ≥ 22–24**	68 (3.3)	2 (2.9)	1 (1.4)	10 (14.5)	1 (1.4)	14 (20.3)	40 (58.0)	9 (13.0)	6 (8.7)	4 (5.8)	2 (2.9)
** ≥ 24–26**	49 (2.4)	1 (2.0)	0 (0)	8 (16.3)	0 (0)	10 (20.4)	23 (46.9)	10 (20.4)	5 (10.2)	1 (2.0)	2 (4.1)
** ≥ 26–28**	19 (0.9)	0 (0)	0 (0)	1 (5.3)	1 (5.3)	1 (5.3)	7 (36.8)	1 (5.3)	4 (21.1)	4 (21.1)	0 (0)
** ≥ 28**	21 (1.0)	0 (0)	1 (4.8)	2 (9.5)	2 (9.5)	2 (9.5)	14 (66.7)	2 (9.5)	2 (9.5)	1 (4.8)	1 (4.8)
**Total**	2062	88 (4.3)	74 (3.6)	116 (5.6)	128 (6.2)	181 (8.8)	833 (40.4)	296 (14.4)	250 (12.1)	214 (10.4)	42 (2.0)

*CS* Cesarean section, *GA* Gestational anemia, *GDM* Gestational diabetes mellitus, *GH* Gestational hypertension, *LBW* Low birth weight, *LGA* Large for gestational age, *MAC* Macrosomia, *PB* Preterm birth, *PROM* Premature rupture of membranes, *SGA* Small for gestational age

**Table 3:** Frequency (n, %) of adverse pregnancy outcomes in different GWG sub-layers in the overweight group

	**Total subjects in sublayers**	**PB** **n (%)**	**LBW** **n (%)**	**MAC** **n (%)**	**SGA** **n (%)**	**LGA** **n (%)**	**CS** **n (%)**	**GA** **n (%)**	**PROM** **n (%)**	**GDM** **n (%)**	**GH** **n (%)**
** < 2**	15 (2.7)	0 (0)	0 (0)	2 (13.3)	1 (6.7)	2 (13.3)	4 (26.7)	1 (6.7)	1 (6.7)	2 (13.3)	0 (0)
** ≥ 2–4**	5 (0.9)	1 (20.0)	1 (20.0)	0 (0)	0 (0)	0 (0)	4 (80.0)	0 (0)	1 (20.0)	1 (20.0)	1 (20.0)
** ≥ 4–6**	21 (3.8)	5 (23.8)	2 (9.5)	1 (4.8)	1 (4.8)	1 (4.8)	10 (47.6)	3 (14.3)	6 (28.6)	8 (38.1)	2 (9.5)
** ≥ 6–8**	33 (6.0)	2 (6.1)	2 (6.1)	1 (3.0)	4 (12.1)	2 (6.1)	17 (51.5)	5 (15.2)	4 (12.1)	9 (27.3)	0 (0)
** ≥ 8–10**	41 (7.4)	1 (2.4)	1 (2.4)	1 (2.4)	3 (7.1)	3 (7.1)	16 (38.1)	5 (11.9)	7 (16.7)	8 (19.0)	2 (4.8)
** ≥ 10–12**	94 (17.0)	4 (4.3)	1 (1.1)	11 (11.7)	1 (1.1)	15 (16.0)	53 (56.4)	17 (18.1)	9 (9.6)	15 (16.0)	2 (2.1)
** ≥ 12–14**	82 (14.8)	5 (6.1)	3 (3.7)	14 (17.1)	3 (3.7)	17 (20.7)	46 (56.1)	12 (14.6)	11 (13.4)	10 (12.2)	3 (3.7)
** ≥ 14–16**	92 (16.6)	6 (6.5)	1 (1.1)	8 (8.6)	3 (3.2)	16 (17.2)	54 (58.1)	9 (9.7)	18 (19.4)	13 (14.0)	3 (3.2)
** ≥ 16–18**	55 (9.9)	3 (5.5)	1 (1.8)	8 (14.5)	0 (0)	11 (20.0)	27 (49.1)	9 (16.4)	9 (16.4)	8 (14.5)	2 (3.6)
** ≥ 18–20**	40 (7.2)	3 (7.5)	1 (2.5)	7 (17.5)	1 (2.5)	9 (22.5)	21 (52.5)	6 (15.0)	7 (17.5)	7 (17.5)	2 (5.0)
** ≥ 20–22**	48 (8.7)	3 (6.3)	0 (0)	9 (18.8)	1 (2.1)	15 (31.3)	30 (62.5)	10 (20.8)	12 (25.0)	12 (25.0)	2 (4.2)
** ≥ 22–24**	13 (2.3)	0 (0)	1 (7.7)	2 (15.4)	2 (15.4)	3 (23.1)	8 (61.5)	1 (7.7)	1 (7.7)	0 (0)	1 (7.7)
** ≥ 24–26**	6 (1.1)	0 (0)	0 (0)	2 (33.3)	0 (0)	2 (33.3)	4 (66.7)	2 (33.3)	1 (16.7)	1 (16.7)	2 (33.3)
** ≥ 26–28**	3 (0.5)	0 (0)	0 (0)	2 (66.7)	0 (0)	2 (66.7)	2 (66.7)	2 (66.7)	0 (0)	0 (0)	0 (0)
** ≥ 28**	6 (1.1)	0 (0)	0 (0)	1 (16.7)	0 (0)	2 (33.3)	2 (33.3)	0 (0)	2 (33.3)	0 (0)	0 (0)
**Total**	556	33 (6.0)	14 (2.5)	69 (12.4)	20 (3.6)	100 (18.0)	298 (53.6)	82 (14.7)	89 (16.0)	94 (16.9)	22 (4.0)

*CS* Cesarean section, *GA* Gestational anemia, *GDM* Gestational diabetes mellitus, *GH* Gestational hypertension, *LBW* Low birth weight, *LGA* Large for gestational age, *MAC* Macrosomia, *PB* Preterm birth, *PROM* Premature rupture of membranes, *SGA* Small for gestational age

**Table 4:** The frequency (n, %) of adverse pregnancy outcomes in different GWG sublayers in the obesity group

	**Total Subjects in sub-layers**	**PB** **n (%)**	**LBW** **n (%)**	**MAC** **n (%)**	**SGA** **n (%)**	**LGA** **n (%)**	**CS** **n (%)**	**GA** **n (%)**	**PROM** **n (%)**	**GDM** **n (%)**	**GH** **n (%)**
** < 2**	9 (6.7)	0 (0)	0 (0)	2 (22.2)	0 (0)	2 (22.2)	4 (44.4)	1 (11.1)	0 (0)	1 (11.1)	0 (0)
** ≥ 2–4**	3 (2.2)	0 (0)	0 (0)	0 (0)	0 (0)	0 (0)	1 (33.3)	0 (0)	0 (0)	2 (66.7)	0 (0)
** ≥ 4–6**	3 (2.2)	0 (0)	0 (0)	0 (0)	0 (0)	0 (0)	1 (33.3)	1 (33.3)	0 (0)	0 (0)	0 (0)
** ≥ 6–8**	15 (11.2)	1 (6.7)	0 (0)	3 (20)	0 (0)	4 (26.7)	6 (40.0)	1 (6.7)	1 (6.7)	4 (26.7)	0 (0)
** ≥ 8–10**	15 (11.2)	0 (0)	0 (0)	2 (13.3)	1 (6.7)	2 (13.3)	7 (46.7)	0 (0)	1 (6.7)	6 (40)	1 (6.7)
** ≥ 10–12**	30 (22.4)	2 (6.7)	1 (3.3)	2 (6.7)	0 (0)	5 (16.7)	16 (53.3)	5 (16.7)	3 (10.0)	3 (10)	8 (26.7)
** ≥ 12–14**	15 (11.2)	1 (6.7)	1 (6.7)	3 (20)	1 (6.7)	4 (26.7)	7 (46.7)	1 (6.7)	3 (20.0)	4 (26.7)	4 (26.7)
** ≥ 14–16**	16 (11.9)	1 (6.3)	0 (0)	4 (25.0)	0 (0)	5 (31.3)	10 (62.5)	3 (18.8)	2 (12.5)	7 (43.8)	0 (0)
** ≥ 16–18**	11 (8.2)	2 (18.2)	1 (9.1)	2 (18.2)	0 (0)	3 (27.3)	8 (72.7)	2 (18.2)	1 (9.1)	0 (0)	1 (9.1)
** ≥ 18–20**	9 (6.7)	0 (0)	0 (0)	2 (22.2)	0 (0)	1 (11.1)	5 (55.6)	1 (11.1)	0 (0)	3 (33.3)	0 (0)
** ≥ 20–22**	3 (2.2)	0 (0)	0 (0)	0 (0)	0 (0)	0 (0)	2 (66.7)	0 (0)	0 (0)	1 (33.3)	0 (0)
** ≥ 22–24**	2 (1.5)	0 (0)	0 (0)	1 (50)	0 (0)	1 (50)	2 (100)	0 (0)	0 (0)	0 (0)	1 (50.0)
** ≥ 24–26**	2 (1.5)	0 (0)	0 (0)	0 (0)	0 (0)	0 (0)	2 (100)	0 (0)	1 (50)	0 (0)	0 (0)
** ≥ 26–28**	0 (0)	0 (0)	0 (0)	0 (0)	0 (0)	0 (0)	0 (0)	0 (0)	0 (0)	0 (0)	0 (0)
** ≥ 28**	1 (0.7)	0 (0)	0 (0)	1 (100)	0 (0)	1 (100)	1 (100)	0 (0)	0 (0)	0 (0)	0 (0)
**Total**	134	7 (5.2)	3 (2.2)	22 (16.4)	2 (1.5)	28 (20.9)	72 (53.7)	15 (11.2)	12 (9.0)	31 (23.1)	15 (11.2)

*CS* Cesarean section, *GA* Gestational anemia, *GDM* Gestational diabetes mellitus, *GH* Gestational hypertension, *LBW* Low birth weight, *LGA* Large for gestational age, *MAC* Macrosomia, *PB* Preterm birth, *PROM* Premature rupture of membranes, *SGA* Small for gestational age

### Recommended GWG for the 4 pre-pregnancy BMI groups

Considering the number of pregnant women in each sub-layer of GWG and the lowest occurrence rate of adverse pregnancy outcomes, we made the recommended value of gestational weight gain for the four pre-pregnancy BMI groups. The recommended GWG for underweight pregnant women is ≥ 8 kg and < 12 kg. The recommended GWG for normal weight pregnant women is ≥ 12 kg and < 14 kg. The recommended GWG for overweight pregnant women is ≥ 8 kg and < 10 kg. The recommended GWG for obese women is < 8 kg (Table 5).

**Table 5:** Occurrence rate (%) of participants with adverse pregnancy outcomes in each sublayer among the 4 pre-pregnancy BMI groups

**Subgroup of GWG **(**kg**)	**Number of participants with adverse pregnancy/total sub-layer participants (occurrence rate (%))**			
	**Underweight**	**Normal weight**	**Overweight**	**Obesity**
** < 2**	1/1 (100)	12/14 (85.7)	7/15 (46.7)	6/9 (66.7)
** ≥ 2–4**	0/1 (0)	5//10 (50.0)	4/5 (80)	2/3 (66.7)
** ≥ 4–6**	4/5 (80)	25/38 (65.8)	17/21 (81)	2/3 (66.7)
** ≥ 6–8**	11/13 (84.6)	41/55 (74.5)	28/33 (84.8)	10/15 (66.7)
** ≥ 8–10**	9/21 (42.9)	83/123 (67.5)	29/41 (69)	12/15 (80)
** ≥ 10–12**	24/50 (48)	205/316 (64.7)	69/94 (73.4)	22/30 (73.3)
** ≥ 12–14**	31/58 (53.4)	196/328 (59.8)	63/82 (76.8)	11/15 (73.3)
** ≥ 14–16**	42/82 (50.6)	278/424 (65.6)	68/92 (73.1)	14/16 (87.5)
** ≥ 16–18**	38/64 (59.4)	155/240 (64.6)	41/55 (74.5)	10/11 (90.9)
** ≥ 18–20**	24/42 (57.1)	123/191 (64.4)	34/40 (85)	5/9 (55.6)
** ≥ 20–22**	24/36 (66.7)	114/163 (69.5)	42/48 (87.5)	3/3 (100)
** ≥ 22–24**	11/20 (55)	48/68 (69.6)	10/13 (76.9)	2/2 (100)
** ≥ 24–26**	7/14 (50)	33/49 (67.3)	5/6 (83.3)	2/2 (100)
** ≥ 26–28**	2/5 (40)	13/19 (68.4)	3/3 (100)	0/0 (0)
** ≥ 28**	4/7 (57.1)	15/21 (71.4)	4/6 (66.7)	1/1 (100)

*GWG* Gestational weight gain

### Comparison of overall occurrence rates of pregnant women with adverse pregnancy outcomes under the three recommended GWG criteria

The different recommended GWG criteria are presented in Supplementary Table 1. GWG was categorized into undergainers, appropriate gainers and overgainers in each pre-pregnancy BMI group according to the three recommended criteria (IOM, Japan and China (Occurrence Method)). The occurrence rate of overall and each adverse pregnant outcome among undergainers, appropriate gainers and undergainers groups in each GWG recommended criterion are shown in Table 6. In the appropriate gainers group, the occurrence rate of participants with overall adverse pregnant outcomes was the lowest according to the China (Occurrence Method) recommendation (60.8%), compared with IOM recommendation (63.4%) and Japan recommendation (64.5%). Therefore, the Occurrence Method criteria were selected as the recommendations for total GWG in Chinese women in terms of the lowest occurrence rate of adverse pregnancy outcomes.

**Table 6:** Occurrence rates of pregnant women with adverse pregnancy outcomes among the three recommendations

**Occurrence rates of pregnant women with adverse pregnancy outcomes n (%)**	**China (Occurrence Method)**			**Japan**			**IOM**		
	**Undergainers (*n* = 651)**	**Appropriate gainers (*n* = 706)**	**Overgainers (*n* = 1815)**	**Undergainers (*n* = 116)**	**Appropriate gainers (*n* = 791)**	**Overgainers (*n* = 2265)**	**Undergainers (*n* = 733)**	**Appropriate gainers (*n* = 1262)**	**Overgainers (*n* = 1177)**
**Total**	443 (68.1)	429 (60.8)	1232 (67.9)	80 (69.0)	510 (64.5)	1514 (66.8)	478 (65.2)	800 (63.4)	826 (70.2)
**PB**	55 (8.5)	33 (4.7)	63 (3.5)	13 (11.2)	57 (7.2)	81 (3.6)	60 (8.2)	52 (4.1)	128 (10.1)
**LBW**	41 (6.3)	26 (3.7)	45 (2.5)	12 (10.3)	41 (5.2)	59 (2.6)	44 (6.0)	43 (3.4)	26 (2.1)
**MAC**	23 (3.5)	36 (5.1)	164 (9.0)	1 (0.9)	29 (3.7)	193 (8.5)	24 (3.3)	68 (5.4)	39 (3.3)
**SGA**	52 (8.0)	44 (6.2)	86 (4.7)	16 (13.8)	55 (7.0)	111 (4.9)	57 (7.8)	77 (6.1)	25 (2.1)
**LGA**	39 (6.0)	55 (7.8)	240 (13.2)	5 (4.3)	50 (6.3)	279 (12.3)	43 (5.9)	102 (8.1)	131 (11.1)
**CS**	241 (37.0)	265 (37.5)	823 (45.3)	45 (38.8)	285 (36.0)	999 (44.1)	260 (35.5)	502 (39.8)	48 (4.1)
**GA**	94 (14.4)	97 (13.7)	252 (13.9)	13 (11.2)	112 (14.2)	318 (14.0)	99 (13.5)	186 (14.7)	189 (16.1)
**PROM**	93 (14.3)	84 (11.9)	221 (12.2)	13 (11.2)	108 (13.7)	277 (12.2)	101 (13.8)	140 (11.1)	567 (48.2)
**GDM**	110 (16.9)	72 (10.2)	190 (10.5)	17 (14.7)	116 (14.7)	239 (10.6)	117 (16.0)	52 (4.1)	158 (13.4)
**GH**	12 (1.8)	13 (1.8)	59 (3.3)	1 (0.9)	14 (1.8)	69 (3.0)	12 (1.6)	43 (3.4)	157 (13.3)

*CS* Cesarean section, *GA* Gestational anemia, *GDM* Gestational diabetes mellitus, *GH* Gestational hypertension, *IOM* Institute of Medicine, *LBW* Low birth weight, *LGA* Large for gestational age, *MAC* Macrosomia, *PB* Preterm birth, *PROM* Premature rupture of membranes, *SGA* Small for gestational age

## Discussion

The present cohort study of 3,172 women indicated that the recommendations for total gestational weight gain in a Chinese population are ≥ 8 kg and < 12 kg for underweight women, ≥ 12 kg and < 14 kg for women in the normal weight range, ≥ 8 kg and < 10 kg for women overweight and < 8 kg for obese women. The findings of the present study provide higher-level evidence for obstetricians to make GWG suggestions to pregnant women with different pre-pregnancy BMIs.

At present, most studies [16, 21] in China use the percentile or quartile method to calculate the suitable recommended value for weight gain during pregnancy, and the percentile method requires that all the subjects should be normal people. Although the recommended GWG calculated by taking an absolutely perfect normal population as research subjects has significance in mathematical statistics, it may not be applicable in guiding clinical practice. Health providers probably want to know more about the occurrence of adverse pregnancy outcomes when GWG reaches a certain range to guide the healthcare of pregnant women in the perinatal period. In this study, the Occurrence Method was used to analyze the incidence of adverse pregnancy outcomes in each weight-gain layer. Through comparative analysis, the weight-gain layer with the least rate of occurrence of adverse outcomes for pregnancy was determined as the appropriate recommended value for pregnant women’s weight gain in a specific BMI group. Therefore, this method is more in line with the evidence-based needs of obstetricians and gynecologists when making clinical decisions.

It is reasonable to categorize recommended GWGs according to pre-pregnancy BMI values. Research has found that a low pre-pregnancy BMI is closely linked with probability of developing anemia during pregnancy [31], which is consistent with the findings of the present study (the occurrence of GA reached 12% in underweight subjects). Furthermore, maternal anemia was proven to be linked to poor birth outcome risks in a recent cohort study in India [32], and the risk increased if anemia and underweight were present simultaneously. About 8% of SGA and 6% of LGA were also observed in the underweight group in our study. An individual pregnant patient data meta-analysis suggested that about 24% of complications were attributable to the mother being overweight pre-pregnancy or obese [4]. A recent meta-analysis found a significant correlation between excessive GWG during the first trimester and the risk of developing GDM [33]. In our study, the occurrence of GDM reached 17 and 23% in overweight and obese subjects, respectively. In general, women with GDM had an increased risk of delivering LGA infants [34, 35], and the occurrence of LGA reached 18 and 21% in our overweight and obese subjects, respectively. Therefore, it is recommended that women with a low BMI before becoming pregnant should strive to put on extra weight, while the opposite applies to women with high pre-pregnancy BMIs. China was reported to have the highest CS rate (46.2%) in the World Health Organization Global Survey [36]. It has also been reported that a pre-pregnancy overweight/obesity state was associated with an elevated risk of requiring cesarean delivery [37–40]. In our study, the CS rate reached as high as 54% in the overweight/obesity group. Excessive weight gain during pregnancy caused increased fat accumulation in the birth canal. The birth canal with an increased resistance and decreased muscle contraction could easily induce abnormal labour and dystocia [41], which will lead to the need for a caesarean delivery.

To guide clinical practice, the IOM published guidelines for appropriate weight gain during pregnancy in 2009 [12]. Since these guidelines were developed based on populations in developed countries, they may not be suitable for Chinese women due to different races, regions, physiques and diets and so forth [42]. Our study also indicated higher occurrence of adverse pregnancy outcomes in appropriate weight gain groups, according to IOM criteria. Once again, the findings proved the necessity of carrying out this prospective cohort study in order to provide theoretical reasons for the formulation and improvement of the recommended GWG for Chinese women.

The present study has the following limitations which should be taken into account. First, the sample size may still not be large enough for more stratification, such as age, and may lack power for a robust assessment. Second, it is regrettable that we were not able to examine long-term outcomes of GWG, e.g. maternal postpartum weight retention or the long-term prognosis of infants, as the data were not available. Third, GWG was determined over the entire pregnancy period and not assessed as weekly gains.

## Conclusion

This study provides the optimal values of GWG to minimize the occurrence of undesirable pregnancy outcomes for each pre-pregnancy BMI group in a population of Chinese women.

## Supplementary Information

**Additional file 1: Supplementary Table 1.** Recommended gestational weight gain of different criteria.

## Data Availability

The datasets used and/or analysed during the current study are available from the corresponding author on reasonable request.

## Abbreviations

CPWCS: Chinese Pregnant Women Cohort Study
CPWCS-PUMC: Chinese Pregnant Women Cohort Study-Peking Union Medical College
CS: Cesarean section
GA: Gestational anemia
GDM: Gestational diabetes mellitus
GWG: Gestational weight gain
IOM: Institute of Medicine
LBW: Low birth weight
LGA: Large for gestational age
MAC: Macrosomia
PB: Preterm birth
GH: Gestational hypertension
PROM: Premature rupture of membranes
SGA: Small for gestational age
