# Supplementary material for: Investigation of optimal gestational weight gain based on the occurrence of adverse pregnancy outcomes for Chinese women: a prospective cohort study
Source: Reprod Biol Endocrinol. 2021 Aug 30;19:130. doi: 10.1186/s12958-021-00797-y (PMC8404327; doi:10.1186/s12958-021-00797-y)
Supplement: Supplementary file 1 — Additional file 1: Supplementary Table 1. Recommended gestational weight gain of different criteria. [file 12958_2021_797_MOESM1_ESM.docx]

**Supplementary Table 1.** Recommended gestational weight gain of different criteria

| **Recommended Criteria** | **Gestational weight gain (kg)** | | | |
| --- | --- | --- | --- | --- |
|  | **Underweight** | **Normal weight** | **Overweight** | **Obesity** |
| **IOM** | 12.5 ~ 18.0 | 11.5 ~ 16.0 | 7.0 ~ 11.5 | 5.0 ~ 9.0 |
| **Japan** | 9.0 ~ 12.0 | 7.0 ~ 12.0 | ≤ 7.0 | ≤ 5.0 |
| **China (Occurrence Method)** | 8.0 ~ 12.0 | 12.0 ~ 14.0 | 8.0 ~ 10.0 | < 8 |
